# IMPACT OF RURALITY ON PALLIATIVE CARE–RELATED SERVICE USE: FINDINGS FROM A POPULATION DATASET

**DOI:** 10.1093/geroni/igae098.2219

**Published:** 2024-12-31

**Authors:** Djin Tay, Jia-Wen Guo, Katherine Ornstein, Kline Dubose, Xiaoming Sheng, Lee Ellington

**Affiliations:** University of Utah, Salt Lake City, Utah, United States; University of Utah, Salt Lake City, Utah, United States; Johns Hopkins University, Baltimore, Maryland, United States; University of Utah, Salt Lake City, Utah, United States; University of Utah, Salt Lake City, Utah, United States; University of Utah, Salt Lake City, Utah, United States

## Abstract

Palliative care, including hospice, supports quality of life of individuals living with serious illness. However, residents in rural areas may have limited access to these services. This study examined urban/rural differences in palliative care-related service claims among older adult cancer patients diagnosed with metastatic breast, lung, colorectal, head and neck, bladder, and melanoma cancers. We hypothesized that rural patients would have less palliative care-related service claims. This study used a retrospective population cohort design with cancer registry and state-wide claims data. Data from patients aged 65 and above with a diagnosis of metastatic cancers were analyzed. N=1,798 patients were identified, with n=511 (28%) living in rural or frontier regions. Patients living in rural areas differed from urban patients by cancer types (p<0.006), race (p=0.015), advance care planning (p=0.002), and hospice use (p=0.040). After diagnosis, only 2% of rural patients and 6% of urban patients had advance care planning claims and approximately half had ever received hospice care (54% rural; 49% urban). While insignificant, rural patients had palliative care (35% vs. 31%) and chemotherapy claims (53% vs. 49%) at higher proportions compared with urban patients (p=0.076 and p=0.055 respectively). There were no observed differences in use of life-sustaining and/or cancer treatments. This was one of the few population-based studies examining rural-urban disparities in palliative care-related services. Despite being a clinical guideline and marker of quality of care in advanced cancers, findings imply low uptake of palliative care services overall, suggesting room for improvement for palliative and end-of-life cancer care delivery.

